# Supplementary material for: Electrical programmable multilevel nonvolatile photonic random-access memory
Source: Light Sci Appl. 2023 Aug 1;12:189. doi: 10.1038/s41377-023-01213-3 (PMC10393989; doi:10.1038/s41377-023-01213-3)
Supplement: Supplementary file 1 — Supplemental material [file 41377_2023_1213_MOESM1_ESM.docx]

Supplementary information for Electrical Programmable Multi-Level Non-volatile Photonic Random-Access Memory

Jiawei Meng^1^, Yaliang Gui^1^, Behrouz Movahhed Nouri^2^, Xiaoxuan Ma^1^, Yifei Zhang^3^, Cosmin-Constantin Popescu^3^, Myungkoo Kang^4^, Mario Miscuglio^1^, Nicola Peserico^1^ Kathleen Richardson^4^, Juejun Hu^3^, Hamed Dalir^2^, Volker J. Sorger^1,2*^

*^1^Department of Electrical and Computer Engineering, George Washington University, Washington, DC 20052, USA*

*^2^Department of Electrical and Computer Engineering, University of Florida, Gainesville, FL 32603, USA*

*^3^Department of Materials Science & Engineering, Massachusetts Institute of Technology, Cambridge, MA 02139, USA*

*^4^CREOL, The College of Optics & Photonics, University of Central Florida, Orlando, FL 32816, USA*

*Corresponding Author: *[sorger@gwu.edu](mailto:sorger@gwu.edu)* & *volker.sorger@ufl.edu*

**Note 1: Fabrication detail of P-RAM**

20 nm GSSe thin film layer is deposited by using thermal evaporator and a 20 nm layer of ${Al}_{2}O_{3}$ is deposited by using ALD as a protection layer to protect GSSe from further oxidization. Then dual Tungsten Titanium microheater is fabricated in a nanofabrication and imaging center at the George Washington University. 200nm think Tungsten Titanium layer is sputtered as first layer. Then another of 200 nm think Al is deposited over the W/Ti route to decrease the overall resistant, increase the energy share of the micro-heater and to protect W/Ti layer from oxidization. Then a thick 400nm ${Al}_{2}O_{3}$ layer is deposited over the full circuit by using the ALD for final oxidization prevention. Contact pad windows are opened by using oxide layer plasma dry etch for electrical probes to connect with circuit for micro-heaters driving.


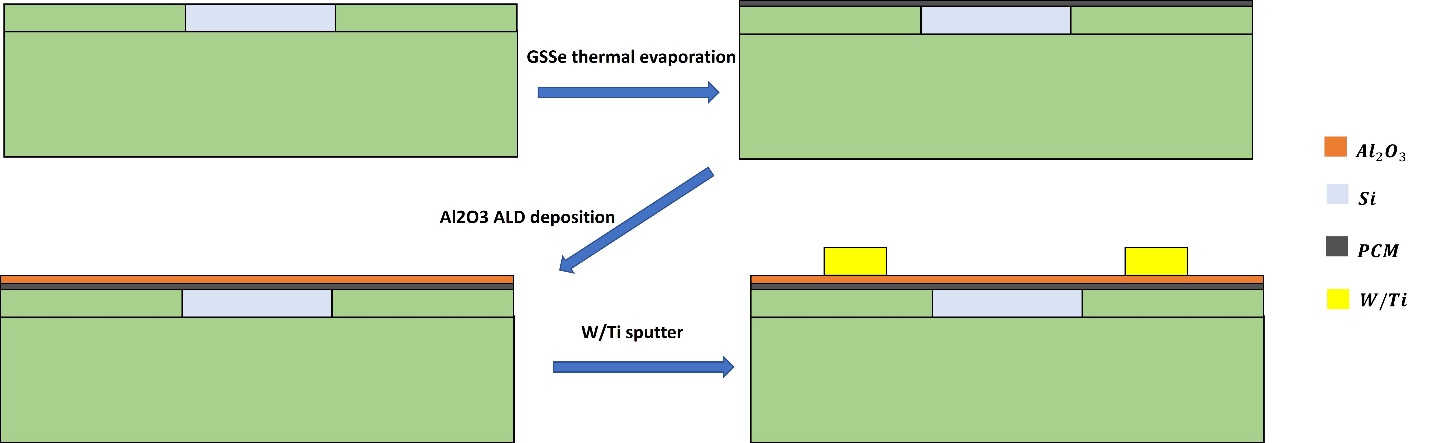


**Figure S1**. Fabrication steps for P-RAM

**Note 2: COMSOL electrical Joule-Heating thermal multi-physics simulation**

The Joule heating process and heat dissipation model were performed using a three-dimensional finite-element method simulation in COMSOL Multiphysics. We used the module of AC/DC Joule-heating module and coupled module of Heat transfer in solids and considered the surface radiation as well as thermal boundary resistance. We coupled this module with the Heat Transfer in Solids, where surface-to-surface radiation and thermal boundary resistance were considered.

**
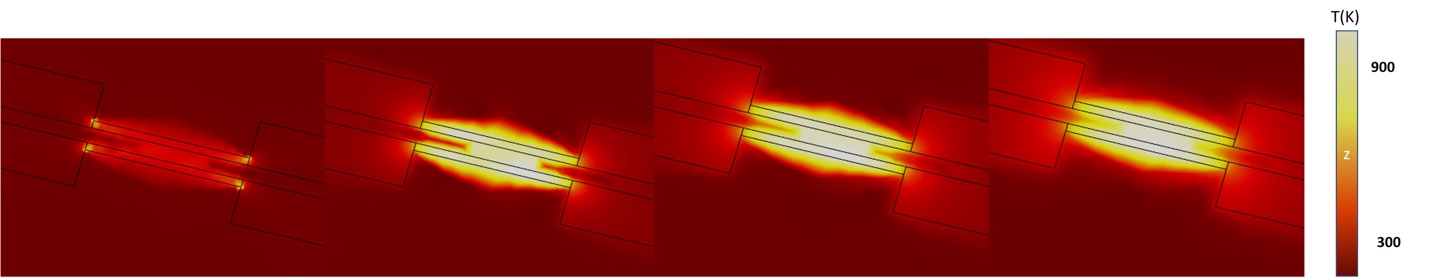
**

**Figure S2.** Heat distribution with the different electrical energy applied to the dual heaters. From left to right, a 2V pulse is applied to the heater with 1/1.5/2/2.5 $\mu s$ pulse width.

**Note 3: COMSOL 2D mode simulation**

COMSOL 2d mode simulation was performed to analysis the effective mode index of active region with bistate of GSSe over the planarized waveguide. The unit length absorption was calculated based on the effective imaginary refractive index from the simulation.


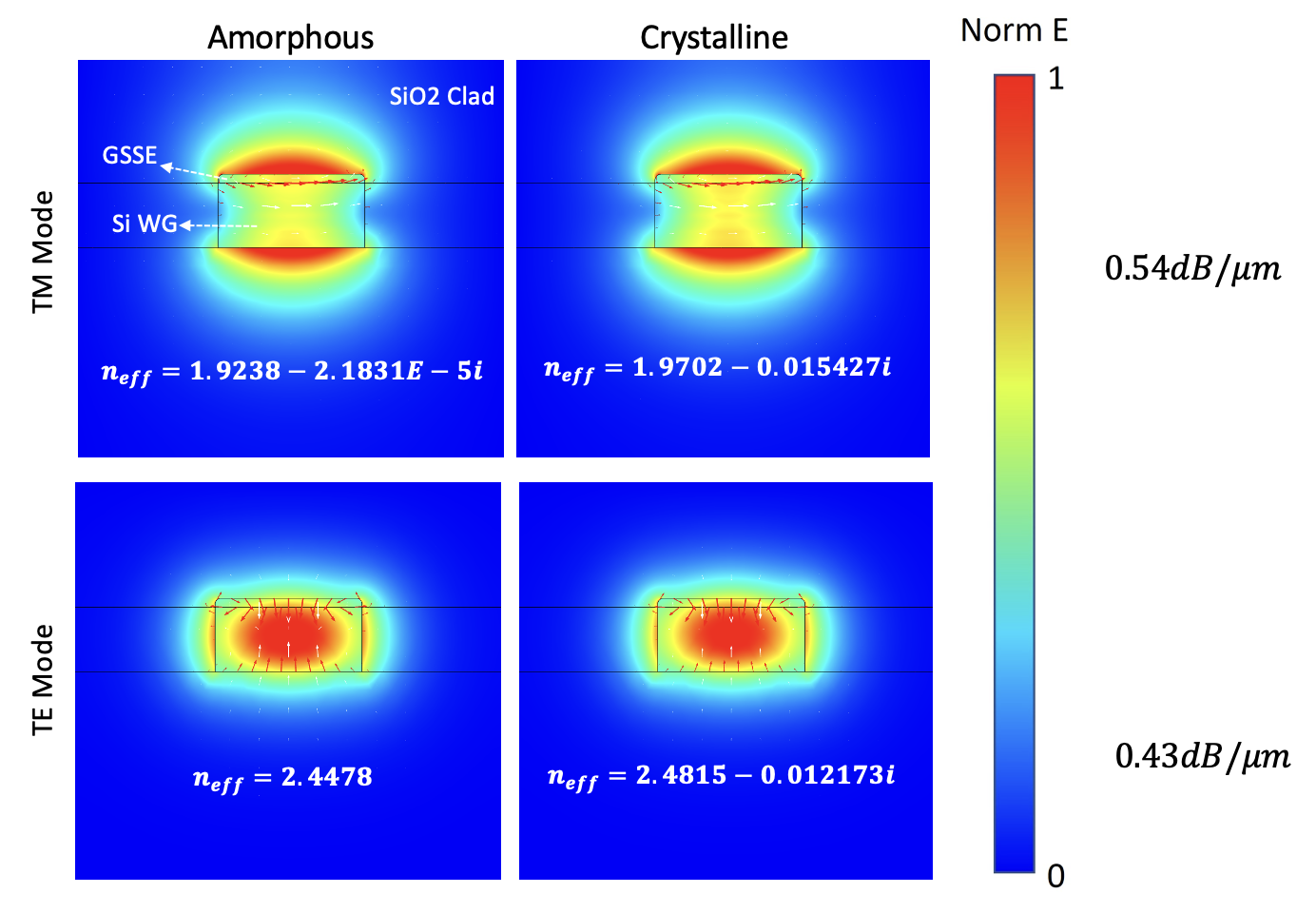


**Figure S3.** 2D mode profile of GSSe over the waveguide.

**Note 4: W/Ti micro-heater structure and schematic optimization for high order of ser-rest cycles**

Two layers of metal was used for the routing of heater along with contact pad. A thick layer of Aluminium was deposited over the based layer of Tungsten/Titanium while the heater section is only one single layer of W/Ti. Then a thick layer of ${Al}_{2}O_{3}$ deposited over the heater with ALD machine.

**
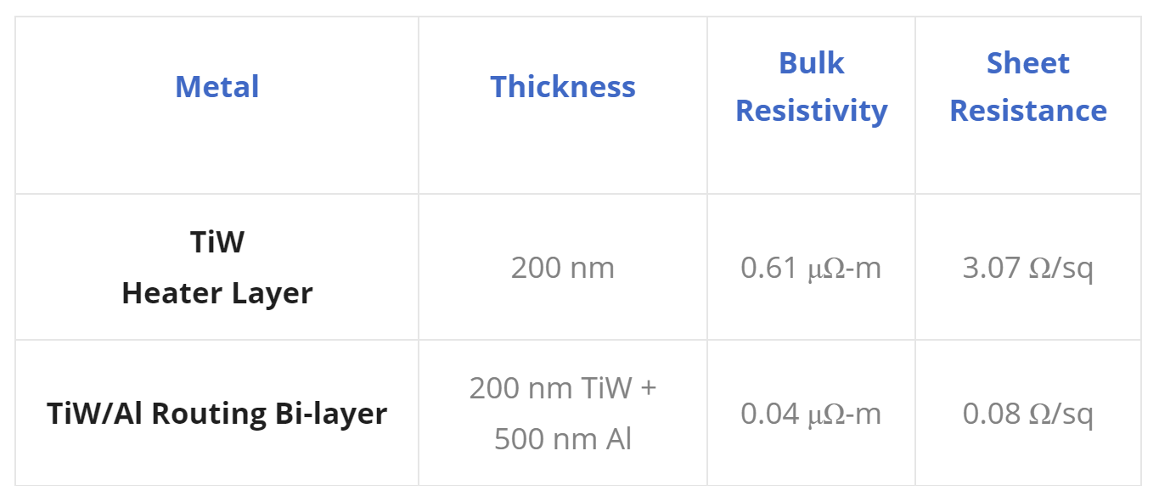
**

The use of two metals increases the electrical efficiency of the devices, as most of the heat generation can be targeted to a specific area of the chip. Using a bi-layer for the routing layer ensures good electrical contact between the routing layer and the heater layer, reduces the contact resistance, and improves uniformity. Moreover, the bi-layer structure in routing helps to reduce the system resistance and increases the voltage share of heater which ultimately increase the energy efficiency. The oxide layer over the heater we deposited works as the protection layer for the heater avoiding break down and increase the lifetime. As the ${Al}_{2}O_{3}$ prevent metal oxidization when it works in high temperature and prevent metal heater physically breaks done when there is a rapid large temperature change with high order cycles.

**Note 5: Measurement set-up**

A maple leaf wafer-scale automated photonic testing system was used to perform the optoelectronic measurements. The high voltage electrical pulses and crystallization electrical pulses were generated with an analog MHz, 16 V pulse generator (National instrument, PXIe-1092). Thorlabs, RXM40AF high speed photodetector was used for the P-RAM speed response measurement.


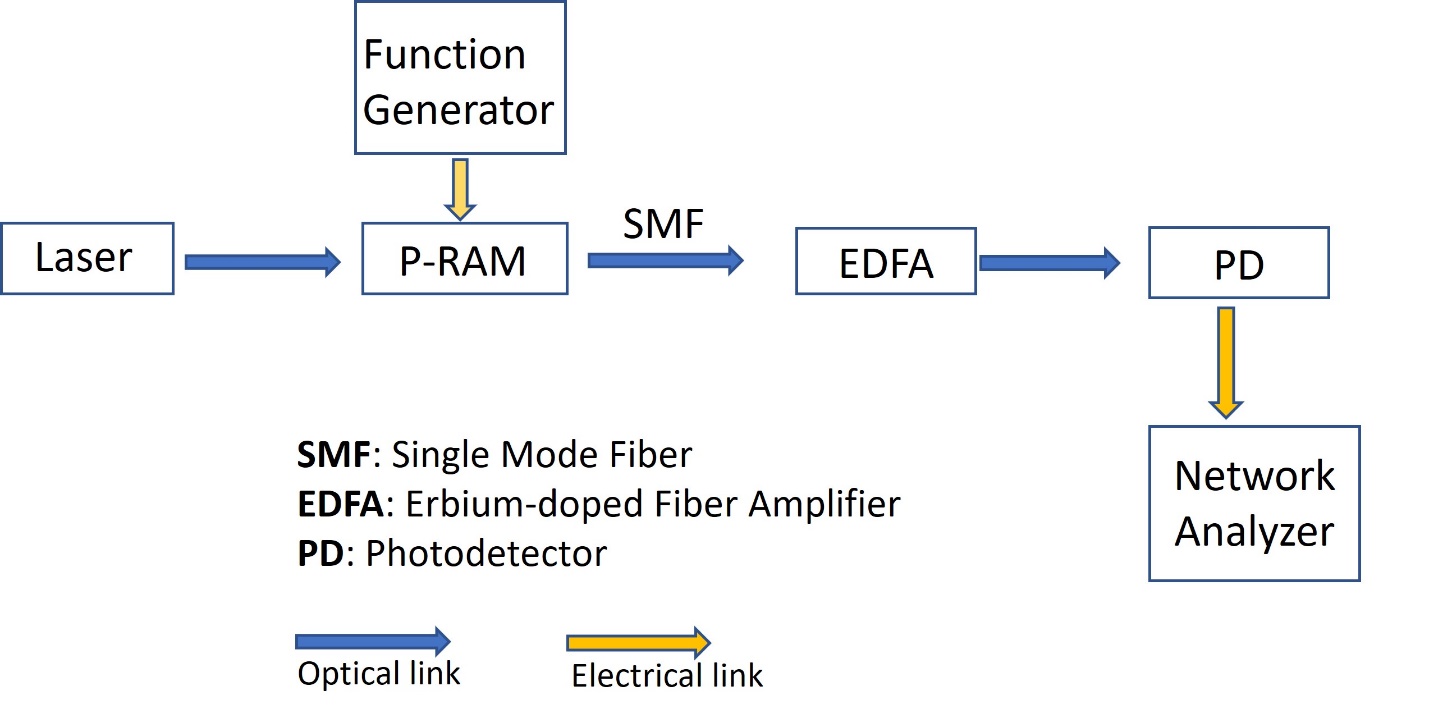


**Figure S4.** Measurement setup

**Note 6: GSSe material process**

Thermal evaporator was used for the deposition of GSSe thin film over the silicon waveguide. A heating up stage (4 minutes in our case) at a lower voltage is required than what normally use for deposition and then a ramp up to deposition temperature / voltage. In our case, our equipment reports a percentage of power so go from 6.3% to around 8 – 9% between the heating stage and the deposition stage. This may have to be tuned though. From the QCM, we try to keep the deposition rate between 8 and 9 A/s and the pressure around 3E-6 Torr before depositing and around or (hopefully) below 8E-6 Torr during the deposition.

**Note 7: GSSe bi-states Bandgap Derivation**

The bandgaps of both amorphous and crystalline GSSe were obtained from the Gen-Osc model used to fit in our own ellipsometry measurement model. We plot the absorption coefficient vs. introduced energy in eV as shown in Fig S3. which indicates the band gap energies of 1.24 eV and 0.81eV for amorphous and crystalline states respectively.


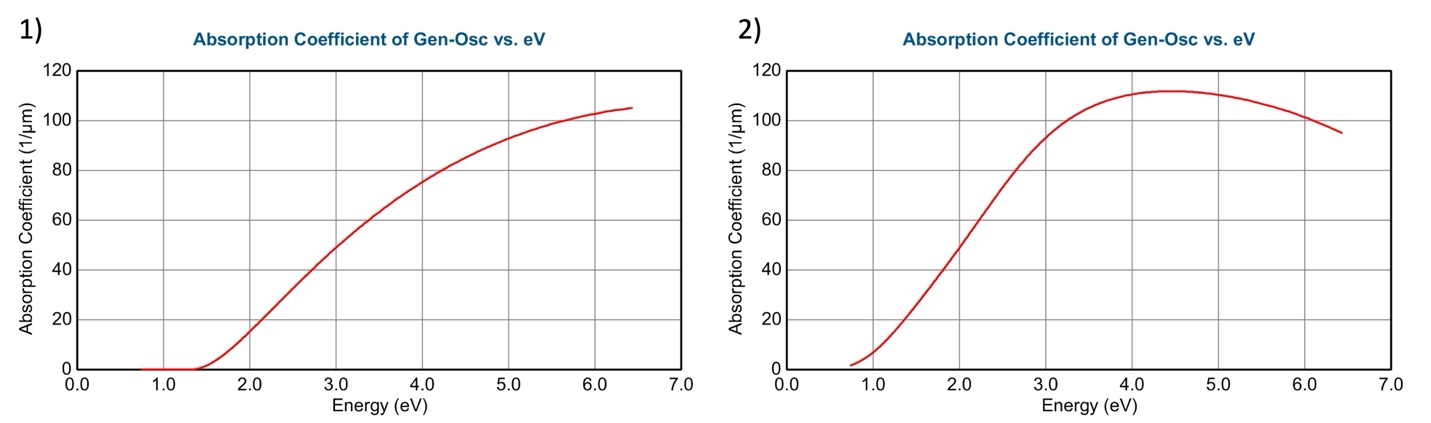


**Figure S5.** (1) Plot of amorphous and (2) crystalline state of GSSe.

**Note 8: 3D mode simulation for how position of dual metal heater influences the effective mode of waveguide.**

The horizontal position between waveguide and metal heaters in double sides was swept from 0nm to 500 nm. The effective absorption coefficient k is shown in Fig. The ideal position for the heater is to be as closer to the waveguide as possible to increase the thermal efficiency while no extra loss will be introduced by the metal. Based on simulation results, 500 nm distance is the sweet spot for the heater position.

**
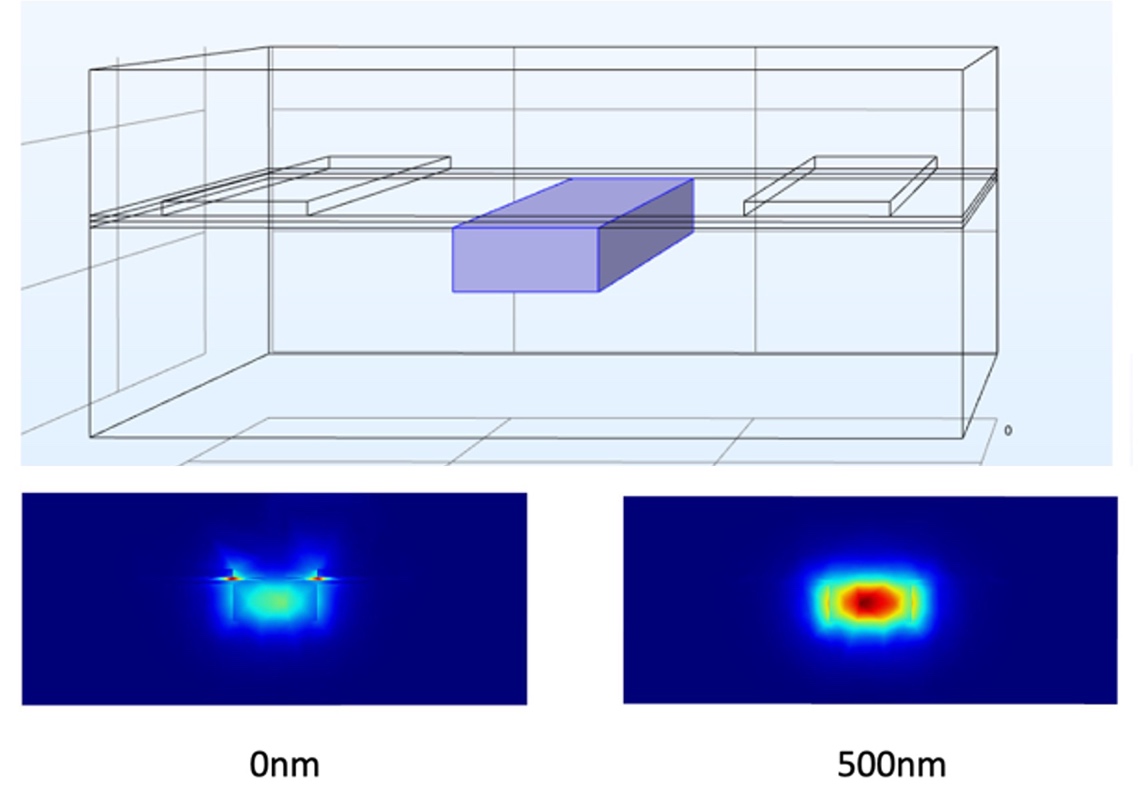
**

**Figure S6.** 3D mode simulation to optimize the position between metal heaters and waveguide.

**
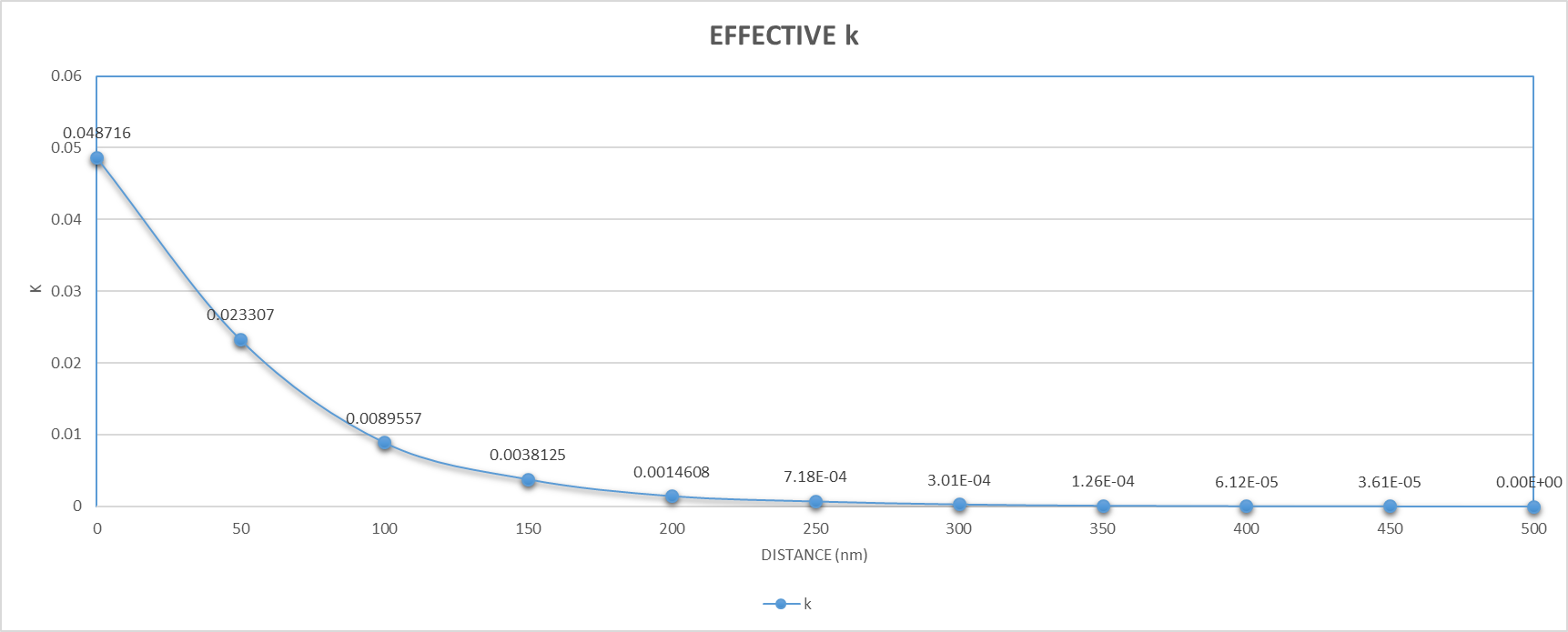
**

**Figure S7.** The simulated effect k vs distance between heater and waveguide.

**Note 9: Supplemental figures.**

**
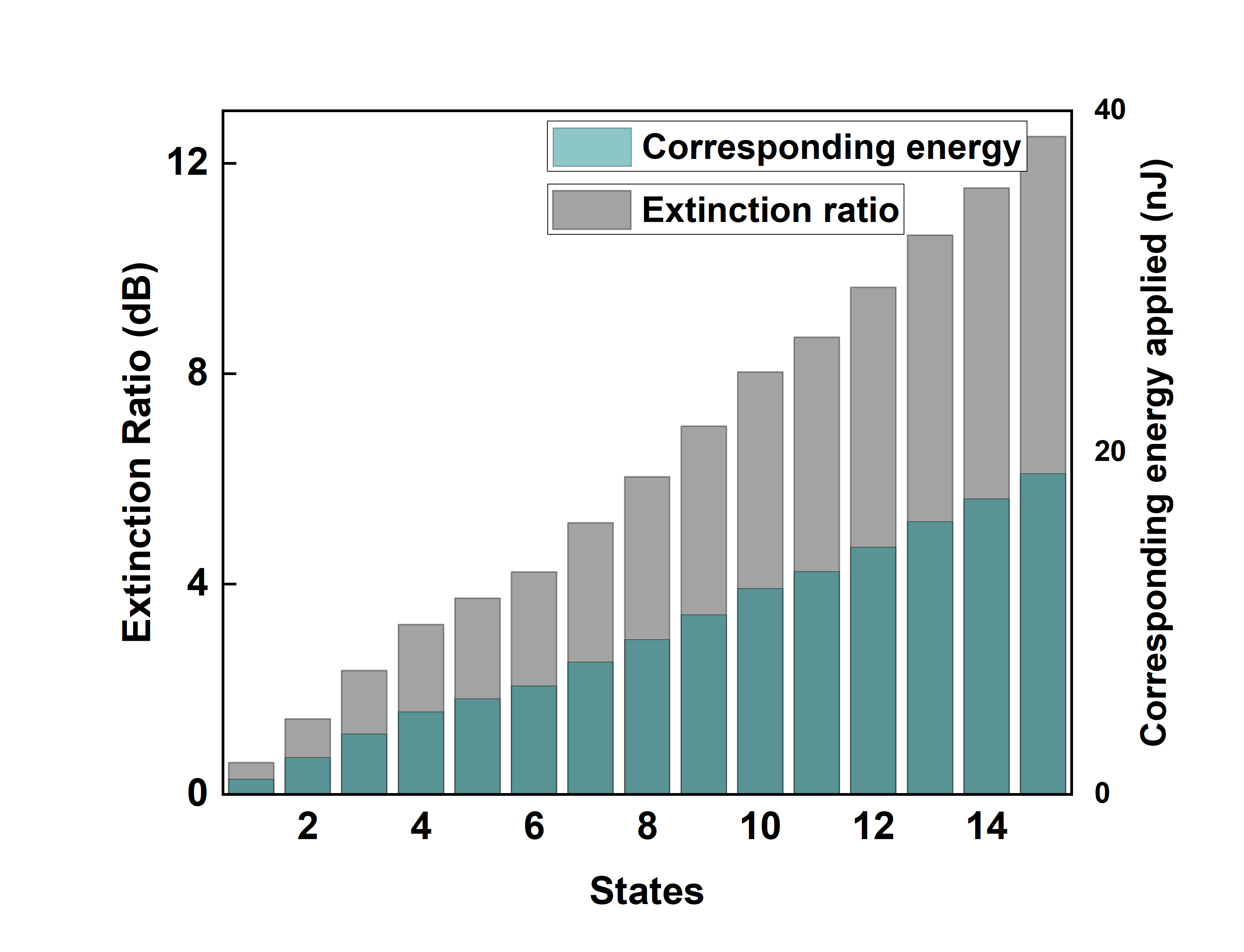
**

**Figure S8.** Optical power response for a 4-bit photonic memory as a function of digital states, for an increasing number of crystalline-wire the Extinction Ratio (ER) increases linearly and uniformly. As well as corresponded total applied energy for each bit.

**Figure S9.** Number of counts agains different optical power response of crystaline during the cyclibility test.

**Note 10: P-RAM key properties summary.**

**Bias condition:** To change the GSSe from amorphous to crystaline, a sequence of pulses, 8 V 2 us width, as shown Fig 3c) will be applied through a micro-heater.

To change the GSSe from crystaline to amorphous state, a single 12 V pulse will be applied through a micro-heater.

**Lifetime:** Until currently, total half million set-reset cycles have been conducted and further cyclability test is still going on.

**Number of bit per cell:** There is one fixed performance value, the unit extinction ration of GSSe is 0.2 dB$\mu m-1$. The number of bit per cell will be determined by the length of GSSe material along the waveguide and minimum step size (dB step-1) set.

**Geometric properties:** As shown in Fig 1g) of main text, 20nm GSSe thin film layer is deposited by and a 20 nm layer of ${Al}_{2}O_{3}$ is deposited as a protection layer. Then dual Tungsten Titanium microheater is fabricated. 200 nm think Tungsten Titanium layer is sputtered as first layer. Then another 200 nm think Al is deposited over the W/Ti route.
